# Persistent Headaches, and How to Cure Them

**Published:** 1890-01

**Authors:** Julian J. Chisolm

**Affiliations:** Professor of Eye and Ear Diseases in the University of Maryland, and Surgeon-in-Chief to the Presbyterian Eye, Ear and Throat Charity Hospital of Baltimore City.


AMERICAN JOURNAL

—OF—

DENTAL SCIENCE.

VOL. XXIII. BALTIMORE, JANUARY, 1890. NO. 9.

ARTICLE I.

### PERSISTENT HEADACHES, AND HOW TO CURE THEM.

BY JULIAN J. CHISOLM, M. D.

[Professor of Eye and Ear Diseases in the University of Maryland, and Surgeon-in-Chief to the Presbyterian Eye, Ear and Throat Charity Hospital of Baltimore City.]

Scientific truths disseminate themselves very slowly. The well-tried and thoroughly proven have to be often discussed, and are as frequently forgotten, before they take root. They then give evidences of the fruit which they should have earlier borne. In this category is placed the medical and now well established fact, that among the many causes of most persistent headaches, eye faults are perhaps the most common. As a rule, they are the *last* to be recognized.

*Headache* is acknowledged to be one of the most constant effects of systemic disturbances. It is present in all of the acute inflammatory diseases, and in most of the chronic ones. When any of the important organs of the body get out of order, the head suffers. The brain, the heart, the stomach, the liver, the kidneys, the uterus, all of them, can produce headache. We know with what readiness the emptying of an impacted bowel will clear up head discomforts. *The mistake made is to believe these exclusive causes of headache.*

Many of these sources of trouble to the head are fortunately readily found, and some of them are very easy of detection. But there is a very large number of head discomforts in which no acute inflammatory condition exists, to explain the disturbance of this sensitive centre. The general health of the individual is good. No one organ seems to be out of order. The digestive apparatus is in perfect condition. The liver is doing its best work. There is no uncomfortable heart throbbing nor face flushings. The kidneys are sound, with normal urine. If there be a uterus, its presence is known only at the catamenial periods. Yet the head is not comfortable.

That supposed omnipresent cause of general disturbance, *Malaria*, is then invoked to account for the frequent complaining. It is found without seeking for it, and it is exercised by frequently repeated doses of quinine, ***accompanied by rest.*** Relief comes temporarily under this treatment, as it would under any other, but the cure is only of short duration. When the malarial theory fails and some other has to be sought, too close confinement to the house or business is suspected, and relaxation from work with fresh air exercise is enjoined. Good at once shows itself from this course of hygiene: but the head discomforts will return.

Then comes suggestive thought on the part of the troubled family physician in his vain efforts to find a satisfactory cause for his patients' sufferings. He calls the trouble ***neuralgia.*** Under this name the patient is often willing to suffer, obtaining occasional relief by taking quinine, morphia, antipyrin, the bromides, or some allied drug. Finally it dawns upon the patients that when they took medicines or open air exercises, and *stopped work*, relief came ; but that when they resumed their occupations of reading, writing or sewing, these being the every day employments of the majority of the population in cities, their heads would ache. From their own observations they would propose the question to their medical adviser: whether in some way the eyes may not be responsible for much of the trouble which they have so long suffered? Once the discovery is made, the cause and effect can be constantly traced. They then find out that they can read, write, or sew themselves into a headache. This sequence is so common, the degree of discomfort being in proportion to the nervous temperament of the patient, that it ought to have been detected at a much earlier period.

*It is a safe rule for a physician to follow: to look to the eyes as a common cause of head disturbances when the frequent headaches of a patient are not produced by some tangible disease.* As a cause of headaches, eye troubles should always precede malaria, neuralgia, biliousness, dyspepsia, and such commonly attributed, but very obscure, sources of disorders; and this even when the patient's painful head symptoms temporarily yield to quinine, iron, arsenic, caffeine, a blue pill, or soda mint.

Unfortunately, when the fault in the eye is paining the head, and is secondarily disturbing the whole body, the eye itself does not necessarily take on a conspicuous congestion. It is not even always painful: so that the casual observer can see in it no cause for trouble. Many persons know that under the continued use of the eyes the head aches, but the seat of pain need not necessarily be in the eyes themselves. Fortunately, in the majority of cases it is the pain in the eyes that precedes the pain in the head. If this sequence always existed there would be less trouble in making a correct and early diagnosis. A very misleading fact with pain-making eyes is, that often vision is apparently perfect. Persons will remark that they can see as far as any one, in evidence that their eyes cannot be the seat of the disturbance. They do not appreciate that sharp vision is one thing, *easy* as contrasted with *labored* vision quite another. They cannot distinguish between the work done and the exertion needful in its accomplishment until the accumulation of the extra effort induces pain.

Some patients will give this as the history of their troubles. Up to a certain time they had as they supposed the strongest of eyes. They overworked them, and now they will stand no continuous work. A book-keeper properly attributes his annoyance to over night work in getting his books up. A school-teacher found the eyes painful after the examination of an extra number of exercises. A lady had no eye-pains until she forced them on fine embroidery for Christmas gifts. A school-boy traces his eye and head-aches to over ambition in preparing for an examination. A student who had unavoidably lost time, decided to keep up with his class by doing back-work in addition to the days requirement: and to effect this, had to drive his eyes for more hours of the twenty-four than prudence allows. With them all, since the first break-down in their eye-sight, a very few minutes of reading, writing or sewing causes the eyes to water, and burn, and produces a brow stricture with eye pains. Now the head is only comfortable when the eyes are at rest. If eye-work is persisted in the pain extends to the temples, then to the top of the head, and even to the back of the neck. Some refer all the pains to the top of the head, while the back of the head and the neck annoys others the most. In a few even the stomach becomes upset, and nausea follows, not because the liver or the brain is diseased, but as reflected disturbances from the over-taxed eye muscles. One patient complained of a peculiar sensation of constriction over the heart, which accompanied the eye and headaches. As he could bring on this very disagreeable chest-pain by reading, and no chest disease could be found upon the most careful examination, the reflex association from the eye to the heart nerves could be clearly traced.

Nature does wonderful work. The function of any organ is a marvel far beyond our comprehension, and the eye is one of the most intricate parts of the body. While we are lost in admiration over its wonderful mechanism, as an optical instrument, we are aware also of some of its imperfections. We find many human eyes far from perfect. In communities advanced in civilization a perfect eye is rather the exception. Nature may start them right, as is seen among the wild tribes, who have exceptionally good eyes, but no education. School abuses soon upset them. *Near-sightedness is becoming too common an eye trouble, and has as its fruitful source the present forced education of the very young.* In these days there is no play time for children. Lessons in school and studies out of school absorb more than the day-light hours. Brain work is known to be more exhausting than hand work, and young eyes cannot submit to this constant application without injury.

There is a Society protecting children from cruel treatment. It prohibits their employment in factories. *The factories which should head the list as most abusive to their general well-being are the schools as they are now conducted.*

The average citizen restricts his idea of muscular labor to the hands alone, not knowing that eye-work is a perpetual muscular labor, and of a very fatiguing character. When a person is reading, or sewing, or writing, the small, but all important eye muscles, are as hard at work in their way as the arm muscles would be in sawing wood, or lifting heavy weights. These eye muscles are as capable of being fatigued, and when fatigued cause pain. This pain need not be restricted to the over-worked muscles themselves. They draw into nervous sympathy contiguous parts of the body, and cause the so-called neuralgias of the head. An extremely common expression, daily received from patients, is that they have so much neuralgia that it makes their eyes pain them. When explained properly it means they have so much eye strain that it makes neuralgia in the head, and then pain in the eyes.

All eye faults (and we use this term as distinct from eye diseases) do not cause headache. By eye faults I mean a faulty form of the eye-ball, a deviation from the perfect type, by which easy focusing of the crystalline lens to form clear pictures on the retina is interfered with. Good sight means an eye which can make sharply defined pictures on the retina for brain interpretation. If this be done without effort the machinery runs smoothly and without discomfort, even if kept up by the hour, or by the day. There is a large class of clear, perfect looking eyes, with apparently excellent sight, which give way under the continued use our every day affairs demand. These eyes would never show their faults were they not pressed by continuous labor. This fault resembles a flaw in the wheel of a railroad car, which can, and has run, for many years at a thirty-mile rate, and seems as good as the best, but when driven at a sixty-mile speed goes to pieces. The trouble with such eyes began when additional work was put upon them, and they have given trouble ever since.

The true headache eye is known as an *astigmatic* one, from the Greek word *a stigma*, not a point, which means that a point of concentrated light is not being clearly made on the retina by the focusing apparatus. This astigmatism is usually a fault in the shape of the cornea, although it may be more rarely produced in the crystalline lens, or might even be from irregularities in the surface of the retina itself. It disturbs the making of sharp retinal pictures, and, to effect a designed purpose, demands on the part of the eye muscles more work than nature intended. Should we similate the cornea to the glass which covers the face of a watch, we can readily understand how the glass may be so squeezed out of shape by lateral pressure as not to fit the perfect rim into which it should adjust itself. In examining such a faulty watch-glass we will find that the curvatures are not uniform in all directions; also that in the direction of the pressed edges the curvature is greater than the opposite or true surface of the watch-glass. The cornea is in a similar manner distorted into astigmatism. When the two meridians of curvature at right angles to each other are taken, they are not found uniform. They should in good eyes represent curved surfaces, all of which are made by one radius. Unfortunately, in astigmatism there is a different radius for each of the two curved surfaces.

The fixed law of optics makes the curvatures of a transparent surface responsible for the focal strength or condensing powers of such a surface. When in an eye these corneal surfaces all correspond, light passing through them on its way to the retina is bent uniformly toward one point, which is called the focus. That brings out on the retina a sharp picture made up of microscopic points of light. If, however, these corneal surfaces are not uniform, the parts corresponding to these varied curvatures, each acting as a lens, will produce two foci of light. Both of these foci cannot be on the same screen at the same time. The perfect part of the cornea will make its image in bright, luminous points on the retina as on a screen. The imperfect part in unfocused luminous rings will cast a shadow over the brighter points, and will more or less blur them. Then comes intuitively an effort on the part of the eye muscles to correct the faulty focusing and make the whole picture bright. This is an unusual muscular act, and has to be repeated for every picture that is made on the retina.

It is easy to calculate how many of these distinct pictures are made in reading or writing, as nearly every letter must have its own and exclusive photographic impression. The brain must register this impression before it is effaced, so that the retina may be made ready for the succeeding letter. To those who have never tried the experiment, it is very startling to discover how small a part of a word we clearly see. Shut one eye and look at a word containing six letters. To see all of them sharply, you will find the eye moving from one side of the word to the other, proving that the six small letters cannot all be focused sharplv simultaneously.

When a well shaped eye adapts itself for reading there is, as it were, a steady contraction of the accommodating muscles, a very easy condition to sustain, even for long periods. With an astigmatic eye a new adjustment of the muscular apparatus has to be made for every picture: a species of perpetual motion, which cannot avoid producing fatigue and consequent pain, both in the eyes and in the head. Each surface has to be separately focused by the eye muscles upon the retina, because the two cannot be focused together. To keep the elasticity of the lens in a perpetual state of activity, focusing first for one corneal meridian, and then for the other, demands incessant activity of the intra-ocular muscles. This tax is especially demanded when the eye is exercised in near work, the more especially under bad illumination. For an astigmatic eye all work is more or less of an effort, and night work by artificial light is especially fatiguing and painful.

The work of the ciliary muscle in astigmatic eyes may be likened to the irregular squeezing of an object by the hand. A well shaped cornea focuses all light passing into the eye by one movement, just as the hand seizes and holds an object by the uniform pressure of all the fingers. No fatigue ensues upon this simple muscular activity. If, however, the fingers do not all clasp simultaneously the object, the thumb and second fingers at one time, to be followed by the remaining fingers in succession, these groups relaxing and contracting at the rate of so many times a minute, fatigue must result. If these irregular muscular hand movements were persisted in, pains would be felt in the fingers, and, extending up the arm, would cause an aching of the whole limb.

The source of sensation for the intra-ocular eye muscles is from branches of the fifth cranial nerve, the great nerve of sensation for the whole head. It is a law of the human economy that when one branch of a sensative nerve is excited all the branches might exhibit sympathetic irritation. From the anatomical distribution of the ophthalmic branch of the fifth nerve to all parts of the scalp we must expect these so-called neuralgic pains to accompany eye irritations: therefore the irregular actions of the eye muscles in astigmatic eyes, being a common cause of local irritations, must be one of the fruitful sources of head disturbances. Then, will all come into play nerve reflexes, intricate and far-spreading, and not restricted to the head alone. Some patients complain of dizziness, giddiness and irregularity in walking. Others of heart irritation, chest constriction, and of nausea. With a few the eye lids are constantly twitching or the face muscles indicate irregular movements. These various symptoms come on as the immediate sequal of eye work. With the very sensitive these uncomfortable results appear when the eyes have been used for only a short time; with others only after long and continuous application. When, however, the eyes finally break down, the irritability persists even when no close eye work is done. Reading, for even a very few minutes, causes pain. The eyes become sensitive to light, and more or less persistent headache results. The irritation excited through the little use of the eyes during the day may last through the night, so that the patient is never freed from head disturbance. With some, these headaches are so constant from the ordinary uses to which the eyes are put, no eye congestion accompanying them, that it is not surprising that the diagnosis is obscure, and that physicians are led astray. I daily see such cases. They report themselves the victims of perpetual neuralgia, or of malarial saturation, or of brain troubles, because the head aches so; or they believe themselves dyspeptic and bilious because of frequent nauseau. With few exceptions they have had years of medical treatment, with only temporary relief when the eyes were rested.

*For such headaches, and they are very common, there are but two remedies.* The natural remedy is to abandon all use of the eyes, and by so doing not excite the irregular fatiguing muscular contractions which the use of astigmatic eyes demands. Simple and effective as this restful idleness is, it is impossible of application. No one in these busy days can make this sacrifice of doing absolutely nothing. The only remedy then left is to correct the excessive and irregular action of the interior eye muscles. This is accomplished by the use of cylinder glasses. When carefully selected, properly mounted and constantly worn, at least for near work, they make all the corneal curvatures act as if uniform in their focusing power; and the function of seeing becomes an easy one.

The observation is constantly made as to the number of young persons now wearing glasses. Every year must increase this ever growing army. Not many years ago the aged alone were supposed to require them, and hence the wearing of spectacles was considered synonymous with being at least, forty years of age. This popular interpretation grated very harshly against the weak points of many a character who would not be thus classified. Notwithstanding the daily observation to the contrary, this conception is still held by many thoughtless people who would rather suffer bodily discomforts than secure immunity from pain at this trivial cost. The simple explanation for the number of spectacles now worn, as seen on the streets, is in the better knowledge of the causes producing the discomforts complained of. For the spectacles with the privileges of full eye work without pain, there was substituted a few years since confinement at home, often to a darkened room, where unseen by the outer world the badly treated patients were forced to abstain from all eye work; and induced by the hope of securing relief, to undergo a persistent, annoying, injurious, because useless, course of medication. The scientific basis for modern medical practice shows an immense advance in this department of surgery.

By wearing proper glasses the eye machinery runs smoothly, and headaches of years continuance disappear as if by magic. A hypodermic injection of morphia cannot more promptly check pain than does the putting on of proper glasses relieve headache. Relief is often immediate with the putting on of cylindrical spectacles, and it becomes permanent under their continued use. Often during the first consultation, when the proper glasses are placed before the eyes for even a few minutes, patients remark *how restful the trial glasses make the head*, and how the old sensations of discomfort come back the moment the glasses are removed.

Quite recently an intelligent lady, 26 years of age, from a distant city, could not for years call a single day's freedom from headache. Very often she had to seek comfort in bed, in a dark room. Her case was one of irregular near sightedness, or, as it is termed " myopic astigmatism." When the proper glasses were adjusted the beauty and comfort of seeing was a revelation to her, which she expressed by face as well as by words. She wore the glasses prescribed, and for the first time in her remembrance enjoyed the comfort of freedom from pain. The giddiness in her case had been so annoying and her gait so unsteady, at times, that she found no pleasure in walking. With the correction of the eye irregularity came a steadiness of tread delightful to enjoy. I found that the nosebridge of her spectacle-frame was a little too narrow, and suggested that the optician open it a little. For three days she had been leading, as she said, a life of luxury, going where she pleased, reading all she wanted, and with perfect bodily comfort. At my suggestion she visited the optician to have the frames properly bent. Awaiting the correction of the frames in the store, she was forced to go without her spectacles for ten minutes, and her headache promptly returned. As she said to me: " You may imagine my anxiety to get my glasses back again, and how I enjoyed them from the moment I put them on."

Astigmatic eyes I see and correct every day.* All astigmatic eyes under continued use cause eye and head pains: headaches more or less persistent being a constant accompaniment of such faultily constructed eye-balls. *Properly selected and properly adjusted cylinder glasses alone will relieve such.* I therefore cure persistent, long-lasting, very annoying and more or less severe headaches every day by prescribing the needed glasses. With very few exceptions all of these patients have been under medical treatment; some for very long periods. With these patients nearly every organ of the body, *except the right one*, has been falsely accused of creating and keeping up the head disturbance. Mercury has been given to many of them for supposed liver torpidity on account of nausea. Iodide of potash has been swallowed by the quantity for the purpose of correcting some supposed syphilitic taint, of which there is no history. During the many years of suffering quinine has been prescribed to the extent of ounces to break up imaginary malarial impressions. The temples have been leeched and blistered, and the head neuralgias have been vigorously, but unsuccessfully, fought by a host of active remedies. Pessaries have been worn because some displacement of the uterus was supposed to be in some mysterious way connected with the head trouble. Often it has occurred, when the physician's long list of remedies is exhausted, that a friend of the patient has suggested that the constant headaches of one of her acquaintances was cured by the wearing of glasses, and perhaps in this case benefit might equally come from their use. In this indirect way some patients finally get relief.

The object of this paper is to draw the attention of physicians to the fact that constant headaches, even when

---

*Nine hundred and ninety-three private patients with astigmatic eyes were treated by me during the year 1889. Several hundred more were treated at the Free Dispensary of the Presbyterian Eye, Ear and Throat Charity Hospital during the same period.

not connected with imperfect vision, or painful eyes, may be caused by faultily constructed eye-balls. Also as irregularity in the focusing power of the eye is a common source of headache, in cases in which the cause of the head disturbance is not patent, its connection with an eye fault ought to be the first eliminated: more especially because when such is the case medication is of no avail in obtaining permanent relief. Tonics to the enfeebled, by increasing muscular strength, may temporarily or partially remove the discomfort. It does not remove the cause, and therefore a tonic cannot cure.

*Rest for such eyes, when pain in the eyes accompany the headache, effects no permanent good.* The trouble remains to annoy as soon as the eyes are again put to use. There is but one way to rest such badly shaped eyes, viz.: correct the fault by properly adjusted glasses. This alone will allow the weary muscles rest.

There are thousands of young persons in our midst to-day who are impatiently resting their eyes for weeks and months at a time, under bad medical advice. Some are kept from school, a serious break in their education. Others can illy spare the time lost from their various pressing occupations. All are taking physic to enable them to escape from annoying headaches which come on whenever they apply themselves. These numerous, long-suffering, and much abused patients need some one to suggest to them the well established fact that, although the eyes do not visibly inflame, they are a common cause of head discomforts: and that this fact in their respective cases is calling for recognition. This advice should come always from the family physician, and should not be accidentally obtained from unprofessional sources.

